# Hepatoprotective Activity of the Total Saponins from *Actinidia valvata* Dunn Root against Carbon Tetrachloride-Induced Liver Damage in Mice

**DOI:** 10.1155/2012/216061

**Published:** 2012-11-06

**Authors:** Liping Qu, Hailiang Xin, Guoyin Zheng, Yonghua Su, Changquan Ling

**Affiliations:** Department of Traditional Chinese Medicine, Changhai Hospital, Second Military Medical University, 168 Changhai Road, Shanghai 200433, China

## Abstract

The protective activity of the total saponins from *Actinidia valvata* Dunn root (TSAV) was studied against carbon-tetrachloride- (CCl_4_-) induced acute liver injury in mice. Mice were orally administered TSAV (50, 100, and 200 mg/kg) for five days and then given CCl_4_. TSAV pretreatment significantly prevented the CCl_4_-induced hepatic damage as indicated by the serum marker enzymes (AST, ALT, and ALP). Parallel to these changes, TSAV also prevented CCl_4_-induced oxidative stress by inhibiting lipid peroxidation (MDA) and restoring the levels of antioxidant enzymes (SOD, CAT, GR, and GPX), GSH and GSSG. In addition, TSAV attenuated the serum TNF-**α** and IL-6 levels and inhibited the serum iNOS and NO levels. Liver histopathology indicated that TSAV alleviated CCl_4_-induced inflammatory infiltration and focal necrosis. TSAV (200 mg/kg) also significantly decreased Bak, Bax mRNA and Fas, FasL, p53, and NF-**κ**B p65 protein expressions and increased Bcl-2 mRNA and protein expressions. Meanwhile, TSAV significantly downregulated caspase-3 and caspase-8 activities and prevented CCl_4_-induced hepatic cell apoptosis. In addition, TSAV exhibited antioxidant activity through scavenging hydroxyl and DPPH free radicals *in vitro*. These results indicated that TSAV could protect mice against CCl_4_-induced acute liver damage possibly through antioxidant, anti-inflammatory activities and regulating apoptotic-related genes.

## 1. Introduction

The liver plays a critical role in regulating several important functions including synthesis, secretion, and xenobiotic metabolism [1]. Liver damage is a widespread pathology which can influence these physiochemical functions and be caused by viral hepatitis, alcoholism, or liver-toxic chemicals, one among them is carbon tetrachloride (CCl_4_). It is metabolized by the P450 enzyme system to yield reactive metabolic products trichloromethyl free radicals, which can initiate the process of lipid peroxidation and contribute to the liver toxicity [2]. Herbal products and traditional Chinese medicine have been widely used for protection against chemical-induced toxicities because of their safety and efficacy. A number of studies have shown that herbal extracts possess antioxidant activity against CCl_4_ hepatotoxicity through reducing oxidative stress and inhibiting lipid peroxidation [2, 3].

*Actinidia valvata* Dunn, affiliated to the genus *Actinidia*, is a shrub mainly growing in eastern China [4] and has a series of applications in traditional Chinese medicine and folk herb. The root of this plant, commonly known as Mao renshen in China, exhibited notable antiinflammatory and anti-tumoral activities and has been used for the treatment of hepatoma, lung carcinoma, and myeloma for a long time [5, 6]. Total saponins of *A. valvata* Dunn root (TSAV) are considered to be one important group of the pharmacologically active ingredients against inflammation and tumor [7, 8]. Recent studies have indicated that some triterpenoids isolated from TSAV showed anticancer activities *in vitro* [9, 10]. However, up to now, the report about hepatoprotective activity of TSAV against liver damage induced by CCl_4_ was not found as far as we know. 

The present study aimed to evaluate the hepatoprotective effects of TSAV against CCl_4_-induced liver damage. The activities of hepatic enzymes in mice were measured and the possible mechanisms of hepatoprotection were also investigated.

## 2. Materials and Methods

### 2.1. Plant Material

*Actinidia valvata* Dunn roots were collected in Changshan County, Zhejiang Province, China, in September 2006, and authenticated by Professor Zheng Hanchen (School of Pharmacy, Second Military Medical University, Shanghai, China). Voucher specimen (no. 20060929) was deposited at Department of TCM, Second Military Medical University.

### 2.2. Chemicals and Reagents

D101 macroporous resin was purchased from Haiguang Chemical Factory (Tianjin, China). CCl_4_ and silymarin were purchased from Sigma Chemical Co. (St. Louis, MO, USA). Diagnostic kits to measure ALT, AST, ALP, TNF-*α*, IL-6, iNOS, NO, MDA, SOD, CAT, GR, GPX, GSH, and GSSG were all purchased from Nanjing Jiancheng Bioengineering Institute (Nanjing, Jiangsu Province, China). DNA ladder extraction kit, caspase-3 and caspaes-8 activity assay kit, Trizol reagent, cell lysis buffer for western blot and IP were purchased from Beyotime Institute of Biotechnology (Nantong, Jiangsu Province, China). Two-Step IHC Detection Reagent was purchased from Zhongshan Goldenbridge Biotechnology Co. Ltd. (Beijing, China). Antibodies against NF-*κ*B p65, Fas, FasL, and GAPDH were supplied by Santa Cruz Biotechnology (Santa Cruz, CA, USA). All other chemicals and solvents were of the high commercially available grades.

### 2.3. Preparation of TSAV from the Medicinal Plant

TSAV was prepared as described by Zheng et al. [11], with minor modifications. The medicinal plant was pulverized into powders (20–40 mesh), and then 600 g of powders were refluxed with 3500 mL of 80% aqueous ethanol for 2 h. The extraction procedure was then repeated twice again. The solvents were evaporated to dryness under vacuum, and the residue was diluted in distilled water. After separating the precipitate by filtration, the remaining solutions were further separated on D101 macroporous resins and eluted with water, 40% aqueous ethanol and 90% aqueous ethanol to produce three fractions. The solutions eluted by water and 40% aqueous ethanol were discarded, and 90% aqueous ethanol solutions were collected and evaporated to dryness under reduced pressure at 50°C. Brown powders (24.34 g) were obtained containing total saponins.

The content of total saponins was determined with Vanillin-HClO_4_ colorimetric method [12]. Quantification was done using external standard calibration, and 2*α*, 3*α*, 24-trihydroxyurs-12-en-28-oic acid was used as the reference standard to calculate the total saponins content. A series of standard of 2*α*, 3*α*, 24-trihydroxyurs-12-en-28-oic acid (ranged from 5 *μ*g/mL–30 *μ*g/mL) was prepared in 80% aqueous ethanol solution to obtain a liner curve with correlation coefficient of 0.9997 (*n* = 6). Absorbance was measured at 550 nm by UV-Vis spectrophotometer (TU-1901, Persee, Beijing, China). The total saponins content was 62.4% (w/w). 

### 2.4. Free Radical Scavenging Activities of TSAV

#### 2.4.1. DPPH Radical Scavenging Assay

The free radical scavenging activity was measured in terms of hydrogen donating or radical-scavenging ability using the stable DPPH radical. Different concentrations of test sample and ascorbic acid were prepared in 80% aqueous ethanol and 2 mL of the sample solution was mixed with 2 mL of 0.1 mg/mL ethanolic DPPH solution. The reaction mixture was shaken vigorously and incubated at 37°C for 30 min. Absorbance was measured at 517 nm using UV-Vis spectrophotometer. The percentage inhibition of the DPPH radical by the samples was calculated using the following equation: inhibition rate (%) = [*A*_0_ − (*A*_1_ − *A*_2_)]/*A*_0_ × 100%, where *A*_0_ is the absorbance of the control, *A*_1_ is the absorbance of the sample and *A*_2_ is the absorbance of the sample, under identical conditions as *A*_1_ with ethanol instead of DPPH solution. Ascorbic acid was used as a reference compound. IC_50_ value (the concentration required to scavenge 50% DPPH free radicals) was calculated. All determinations were performed in triplicate.

#### 2.4.2. Hydroxyl Radical Scavenging Assay

Hydroxyl radical scavenging test was carried out by the described method [13]. The reaction solution was incubated at 37°C for 30 min. Absorbance was measured at 520 nm using UV-Vis spectrophotometer. The inhibition rate was calculated as follows: [(*A*_1_ − *A*_2_)/(*A*_0_ − *A*_2_)] × 100%, where *A*_0_ is the absorbance of the control, *A*_1_ is the absorbance of the sample, and *A*_2_ is the absorbance of the blank sample.

### 2.5. Animals and Experiment Design

Kunming male mice (18–22 g) were obtained from the Sippr-Bk Lab. Animal Ltd. Co. (Shanghai, China) and fed with certified standard diet and tap water *ad libitum*. Temperature and humidity were regulated at 21–23°C and 50–60%, respectively. The study protocol was in accordance with the Guide for the Care and Use of Laboratory Animals as adopted by the United States National Institute of Health and was approved by the Animal Ethic Review Committees of Second Military Medical University.

After one week of acclimatization, the mice were randomly divided into six groups of ten each: (I) control group; (II) CCl_4_ group; (III) low dose group of TASV; (IV) middle dose group of TSAV; (V) high dose group of TSAV; (VI) silymarin group. Groups I and II received 0.5% CMC-Na (200 mg/kg), Groups III, IV, and V received TSAV (50, 100, and 200 mg/kg, resp.) and Group VI received silymarin (200 mg/kg). Drugs and vehicle were administrated (i.g.) once daily for 5 consecutive days. Two hours after the final treatment, the mice in Groups II–VI were treated with 0.3% CCl_4_ (10 mL/kg, i.p., dilution with olive oil), as described previously [14]. Twenty-four hours after administrating CCl_4_, the blood was collected and mice were sacrificed. The collected blood was placed 45 min for clot formation and then centrifuged 4000 ×g for 10 min for separation of serum, which was used for the biochemical analyses. The livers were immediately rinsed with saline, blotted on filter paper, weighed and finally stored at −80°C pending biochemical analyses. One part of liver was cut and put into a flasket containing 10% buffered formalin solution for the following histopathology analyses. 

### 2.6. Serum Biochemical Parameters

The activities of ALT, AST, ALP, TNF-*α*, IL-6, iNOS, and NO in serum were measured by spectrophotometer using enzymatic kits according to the manufacturer's instructions.

### 2.7. Lipid Peroxidation and Antioxidant Enzyme Activities

Hepatic tissues were homogenized in frozen normal saline and centrifuged at 10000 ×g for 10 min. The supernatant was used for the measurement of SOD, CAT, GR, GPX, GSH, and GSSG, which were determined by following the instructions on the commercial kits. Lipid peroxidation was estimated according to MDA diagnostic kits.

### 2.8. Caspase-3 and Caspase-8 Activities

The activities of caspase-3 and caspase-8 were measured strictly according to the instructions of caspase-3 and caspase-8 activity assay kit. Briefly, the mixture of 80 *μ*L detection buffer, 10 *μ*L samples and 10 *μ*L Ac-IETD-*p*NA was incubated at 37°C for 60 min, and OD_405_ was measured. The activities of them were calculated based on the standard curve.

### 2.9. Histology and Immunohistochemistry

Formalin-fixed tissue samples were embedded in paraffin and 5 *μ*m sections were cut. Replicate sections were stained with haemotoxylin and eosin (HE) for observing the liver damage. Additional sections were used for the following immunohistochemistry test through two-step IHC detection. 3% H_2_O_2_ was used to block endogenous peroxidase activity for 10 min and normal goat serum to block nonspecific protein binding for 30 min. After microwave antigen repair, the sections were incubated at 4°C overnight with rabbit anti-Bcl-2 and anti-p53 antibody (1 : 100, dilution), followed by incubation at 37°C in PV6001 for 30 min, and were visualized with 3,3′-diaminobenzidine tetrahydrochloride (DAB) substrate and counterstained by haemotoxylin. Image was taken by inverted digital image light microscopy (Nikon Eclipse TE2000-U, Nikon Instruments Co. Ltd., Tokyo, Japan). The tissues stained brown were considered as positive, and the IOD value was analyzed by Image-Pro Plus software (Media Cybernetics, MD, USA) to evaluate the protein expressions [15, 16].

### 2.10. Protein Extraction and Western Blot Analysis

The liver were washed twice with cold PBS and then lysed in appropriate volume of cold lysis buffer containing 1 mM PMSF lysates which were centrifuged at 12000 ×g for 15 min at 4°C. The total protein was obtained and protein content was determined by coomassie brilliant blue G. Western blot assays were performed as follows: protein (5 mg/mL) was denatured by mixing with an equal volume of 2 × sample loading buffer and then boiling at 100°C for 5 min [17]. An equal protein amount was loaded onto an SDS gel, separated electrophoresis, and transferred to a PVDF membrane. 10% polyacrylamide gel was used for all the electrophoresis. The transmembrane times for NF-*κ*B p65, Fas, FasL, and GAPDH were 50, 45, 35, and 30 min, respectively. After the PVDF membrane was incubated with 10 mM TBS with 1.0% Tween 20 and 5% dehydrated skim milk to block nonspecific protein binding, the membrane was incubated with primary antibodies overnight at 4°C and incubated either rabbit anti-NF-*κ*B p65 (1 : 600 dilution), rabbit anti-Fas (1 : 200 dilution), rabbit anti-FasL (1 : 500 dilution) or mouse anti-GAPDH (1 : 2000 dilution). Blots were then incubated with horseradish peroxidase-conjugated goat anti-rabbit IgG or horseradish peroxidase-conjugated goat anti-mouse IgG for 2 h at room temperature at a 1 : 2000 dilution. Detection was performed by an enhanced chemiluminescence method and photographed by BioSpectrum Gel Imaging System (UVP, Upland, CA, USA). The data were normalized by GAPDH (IOD of objective protein versus IOD of GAPDH protein).

### 2.11. Reverse Transcriptional Polymerase Chain Reaction (RT-PCR)

Trizol reagent was used to prepare RNA of cells. 500 ng of total RNA was required in the following RT-PCR each time. Specific designed primers were shown in Table 1. RT-PCR was performed according to the protocol of Takara RNA PCR Kit and amplified in a TC-512 PCR system (Techne, Barloworld Scientific Ltd., UK). RNA samples were first reverse transcribed and immediately amplified by PCR. The amplification profile was performed by denaturation at 94°C for 1 min, annealing at 60°C, and extension at 72°C for 1 min, and 50 additional cycles were used for amplification. IOD values of the electrophoresis bands were analyzed by BioSpectrum Gel Imaging System.

### 2.12. TUNEL Assay

Apoptosis was detected by TUNEL staining using *in situ* apoptosis detection kit (Roche, Shanghai, China). Paraffin-embedded liver tissues were processed for TUNEL labeling. The images were obtained using fluorescence microscopy (Olympus, Tokyo, Japan).

### 2.13. DNA Ladder

DNA samples were extracted using DNA ladder extraction kit with spin column then were electrophoretically separated in 1% agarose gel and stained with ethidium bromide. The agarose gel was visualized and photographed under ultraviolet light by BioSpectrum Gel Imaging System.

### 2.14. Statistical Analysis

All data were expressed as mean ± standard deviation (SD) and significant difference between the groups was statistically analyzed by one-way ANOVA. A difference was considered significant at *P* < 0.05.

## 3. Results 

### 3.1. Antioxidant Activity of TSAV *In Vitro*

**Figure 1:**
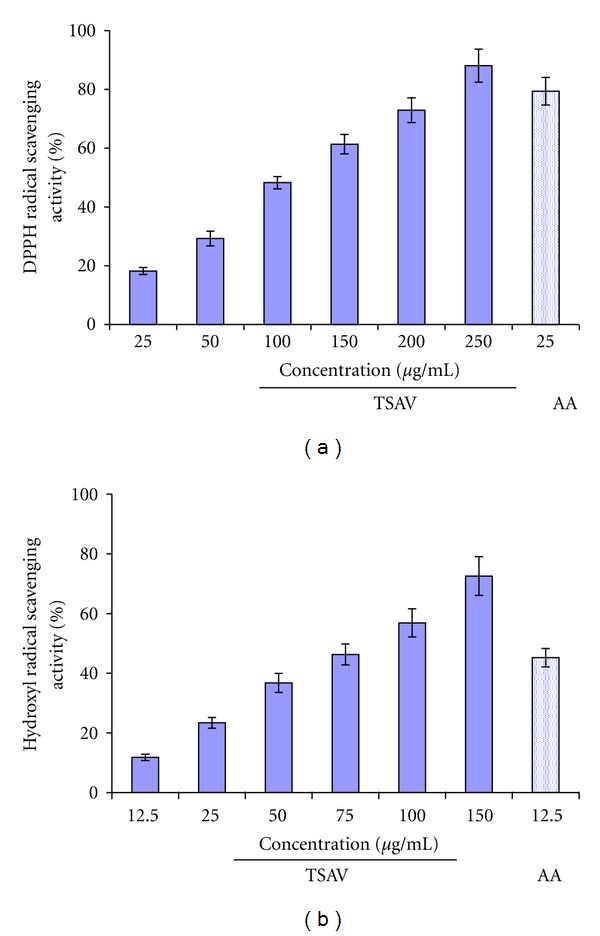
DPPH and hydroxyl radical scavenging activities of different concentrations of TSAV. Ascorbic acid (AA) was used as reference compound. The data are presented as means ± SD, *n* = 3.

 Free radical scavenging effect of TSAV was tested and the results are presented in Figure 1. TSAV in the range 15–250 *μ*g/mL exhibited concentration-dependent DPPH scavenging activity (18.23–88.06% inhibition, IC_50_ 87.78 *μ*g/mL), being less active than ascorbic acid (IC_50_ 12.08 *μ*g/mL). Hydroxyl radical scavenging effect was 36.75%, 46.29%, and 56.87%, respectively, for 50, 75, and 100 *μ*g/mL of TSAV. The IC_50_ value was 74.21 *μ*g/mL and it was compared with ascorbic acid (IC_50_ 20.86 *μ*g/mL) under same conditions. The present study showed that TSAV could effectively scavenge DPPH and hydroxyl radicals.

### 3.2. Histopathology

**Figure 2:**
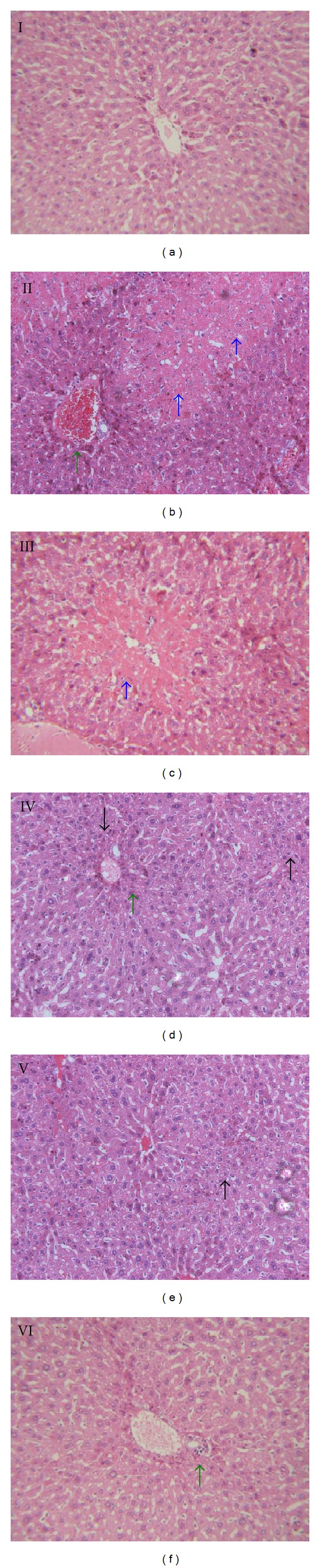
Protective effects of the TSAV pretreatment on CCl_4_-induced liver damage. Histological examination was performed under a light microscope (original magnification: ×100) with HE staining on liver tissues. Group I: control; Group II: CCl_4_; Group III: TSAV 50 mg/kg + CCl_4_; Group IV: TSAV 100 mg/kg + CCl_4_; Group V: TSAV 200 mg/kg + CCl_4_; Group VI: silymarin 200 mg/kg + CCl_4_. Blue, green, and black arrows indicate cell necrosis, inflammatory infiltration, and nuclear pyknosis, respectively.

Histopathological profile of the control mice showed normal hepatocytes with well cytoplasm, prominent nucleus, nucleolus and central vein. There was no sign of inflammation or necrosis in these mice (Figure 2(a)). In mice treated with CCl_4_ only, liver sections showed hepatocyte nuclear pyknosis, hepatic cord degeneration, inflammatory infiltration, and marked necrosis (Figure 2(b)). Pretreatment with TSAV at 50 and 100 mg/kg dose showed reduction of necrosed area and inflammatory infiltrates (Figures 2(c) and 2(d)). TSAV and silymarin at 200 mg/kg dose showed sparse inflammatory cell infiltration and greater reduction of nuclear pyknosis of hepatic cells (Figures 2(e) and 2(f)) as compared with 50 and 100 mg/kg dose. These results indicated that TSAV could ameliorate the severity of liver damage and protect liver from CCl_4_-induced injury effectively.

### 3.3. Serum Enzymes

The effects of TSAV at three dose levels on serum marker enzymes in CCl_4_-induced hepatic injury are shown in Table 2. Serum activities of AST, ALT, and ALP enzymes in CCl_4_ group were significantly increased compared with the control group, which reflected the severity of liver injury. TSAV with different doses and silymarin pretreatments significantly reduced the levels of serum AST, ALT, and ALP as compared with the CCl_4_ group. The effects of TSAV at high dose were found to more markedly reduce their activities than those of silymarin.

### 3.4. Lipid Peroxidation

The effects of different groups on the activities of MDA in the liver are shown in Table 3. CCl_4_ increased the hepatic MDA concentration significantly which was inhibited by TSAV (middle and high dose) and silymarin pretreatment. TSAV at high dose was most effective in inhibiting the hepatic lipid peroxidation than that of silymarin.

### 3.5. Antioxidant Enzymes

Activities of the antioxidant enzymes of hepatic damage: SOD, CAT, GPX, and GR decreased significantly in CCl_4_ group compared with the control group (Table 3). There were dramatic increases in their activities in TSAV (middle and high dose) and silymarin groups compared with the CCl_4_ group, and antioxidant effects of TSAV pretreatments at high dose were better than those of silymarin.

### 3.6. Hepatic GSH and GSSG Levels

CCl_4_ treatment caused a significant decrease of GSH and increase of GSSG compared with the control group. TSAV pretreatment prevented these changes more effectively than that of silymarin. GSH/GSSG ratio was significantly reduced in the CCl_4_ group compared with the control group, while TSAV prevented this effect (Table 3).

### 3.7. Serum TNF-*α*, IL-6, iNOS, and NO Levels

**Figure 3:**
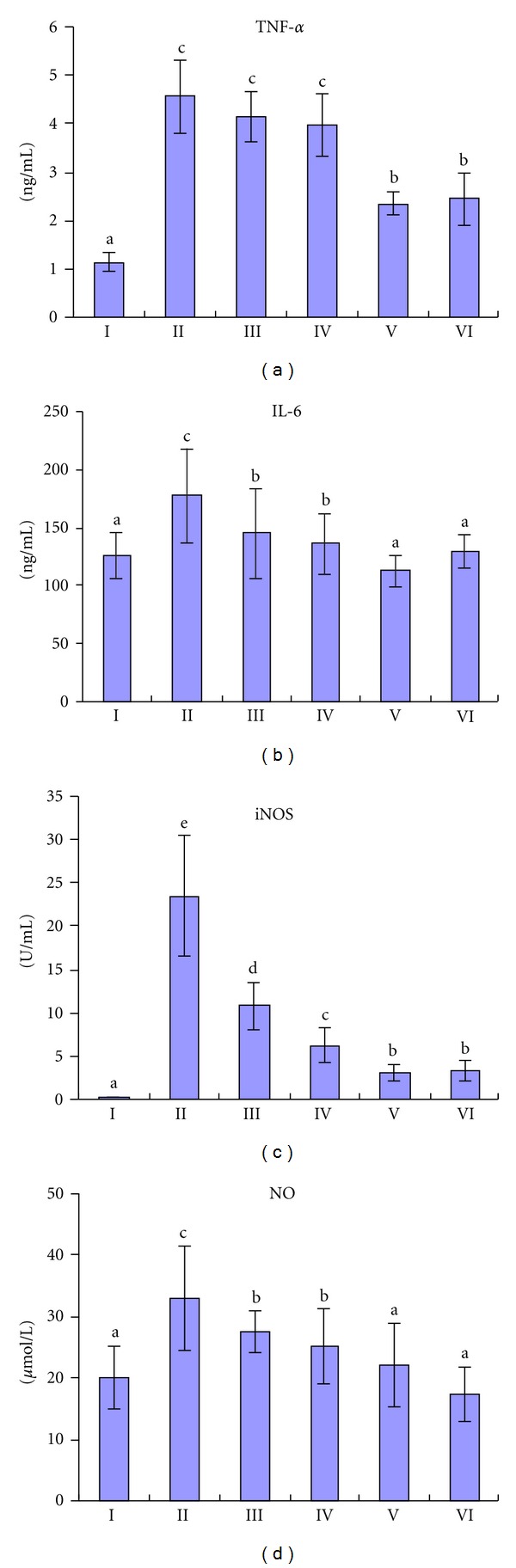
Effects of different concentrations of TSAV on the serum TNF-*α*, IL-6, iNOS, and NO activities. Group I: control; Group II: CCl_4_; Group III: TSAV 50 mg/kg + CCl_4_; Group IV: TSAV 100 mg/kg + CCl_4_; Group V: TSAV 200 mg/kg + CCl_4_; Group VI: silymarin 200 mg/kg + CCl_4_. Each bar represents the mean ± SD, *n* = 10; bars with different alphabets differ significantly at *P* < 0.05 level.

CCl_4_ treatment significantly increased the serum TNF-*α*, IL-6, iNOS, and NO levels in the mice liver. There were marked decreases of TNF-*α* level in high dose TSAV group and of NO level in the middle and high dose TSAV group, and there were dramatic decreases of iNOS level and IL-6 level in the three TSAV groups compared with the CCl_4_ group (shown in Figure 3). Moreover, the effects of TSAV at high dose were found to more markedly reduce the levels of TNF-*α* and IL-6 than those of silymarin.

### 3.8. Caspase-3 and Caspase-8 Activities

**Figure 4:**
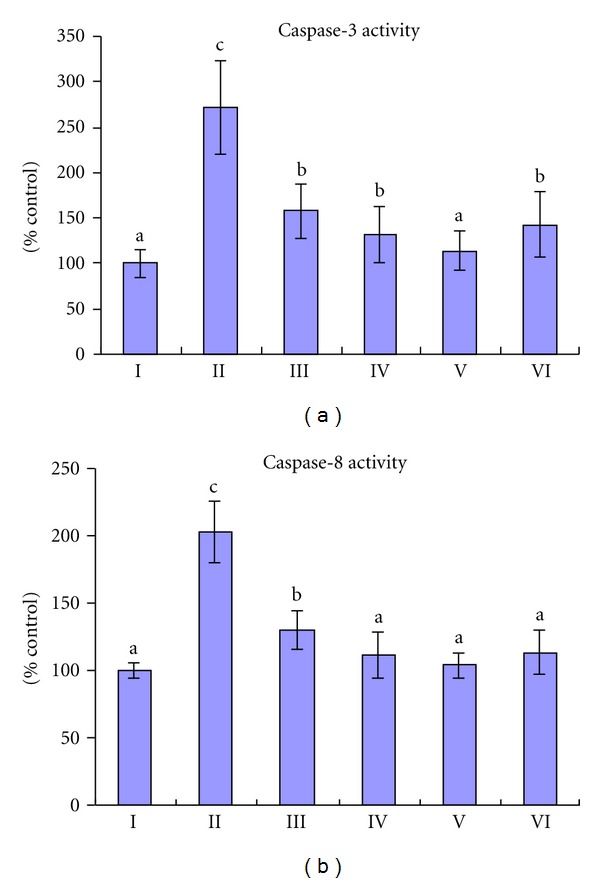
Effects of different concentrations of TSAV on the caspase-3 and caspase-8 activities. Group I: control; Group II: CCl_4_; Group III: TSAV 50 mg/kg + CCl_4_; Group IV: TSAV 100 mg/kg + CCl_4_; Group V: TSAV 200 mg/kg + CCl_4_; Group VI: silymarin 200 mg/kg + CCl_4_. Each bar represents the mean ± SD, *n* = 10; bars with different alphabets differ significantly at *P* < 0.05 level.

The activities of caspase-3 and caspase-8 in the CCl_4_ group were significantly increased. TSAV pretreatment at three doses significantly decreased their activities (shown in Figure 4). The activities of caspase-3 and caspase-8 in high dose of TSAV were reduced by 58.08% and 48.77% compared with the CCl_4_ group. 

### 3.9. TUNEL Assay and DNA Ladder

**Figure 5:**
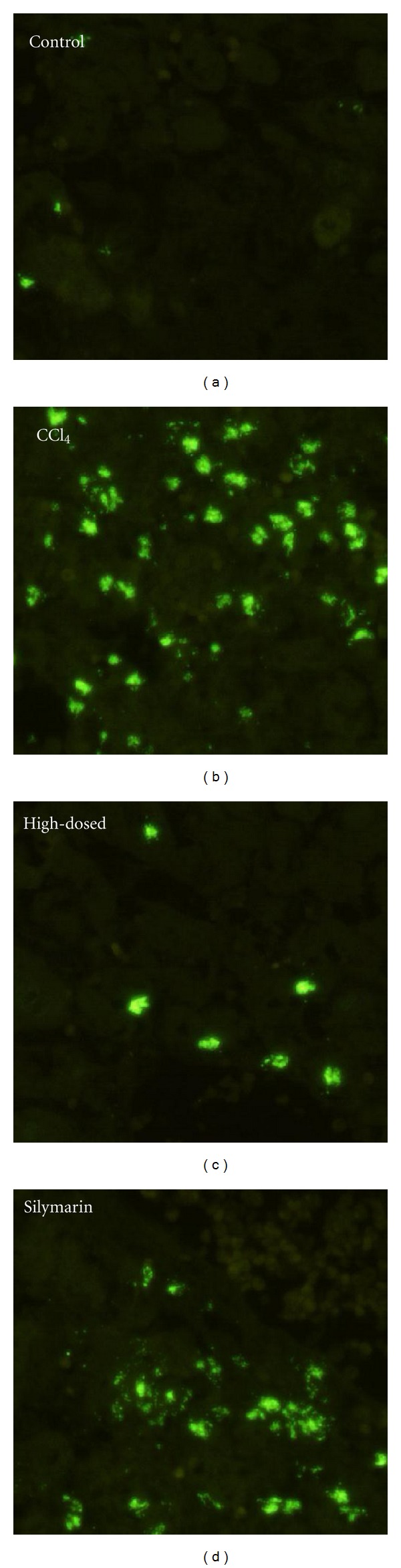
TUNEL stained histological examination was performed under fluorescence microscopy on liver tissues (original magnification: ×100). CCl_4_: showing a large number of TUNEL-positive cells; high-dosed: TSAV 200 mg/kg + CCl_4_, showing only a few TUNEL-positive cells; Silymarin: silymarin 200 mg/kg + CCl_4_, showing a few TUNEL-positive cells.

**Figure 6:**
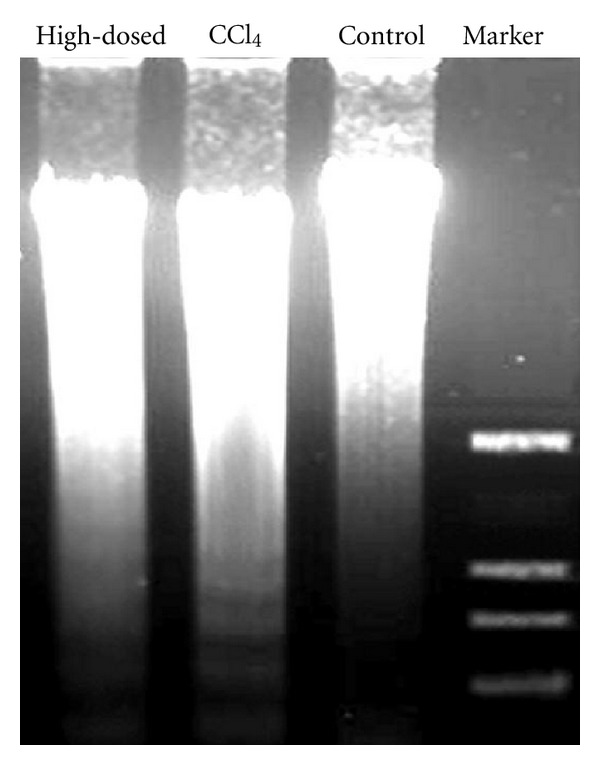
DNA laddering was electrophoresed on 1% agarose gels. High-dosed: TSAV 200 mg/kg + CCl_4_.

An *in situ* cell apoptosis detection kit was used to assess apoptosis in liver tissues. As shown in Figure 5, the number of TUNEL-positive apoptotic nuclei increased after CCl_4_ treatment, and few TUNEL-positive cells were observed in the livers obtained from mice pretreated with TSAV (200 mg/kg) and silymarin. In addition, the effect of TSAV on DNA fragmentation was also investigated (Figure 6). The characteristic DNA ladder was found in the CCl_4_ group; however, the DNA ladder was significantly attenuated in TSAV high-dosed group, which indicated that TSAV could inhibit hepatocyte apoptosis induced by CCl_4_ in mice.

### 3.10. Western Blot Analysis

**Figure 7:**
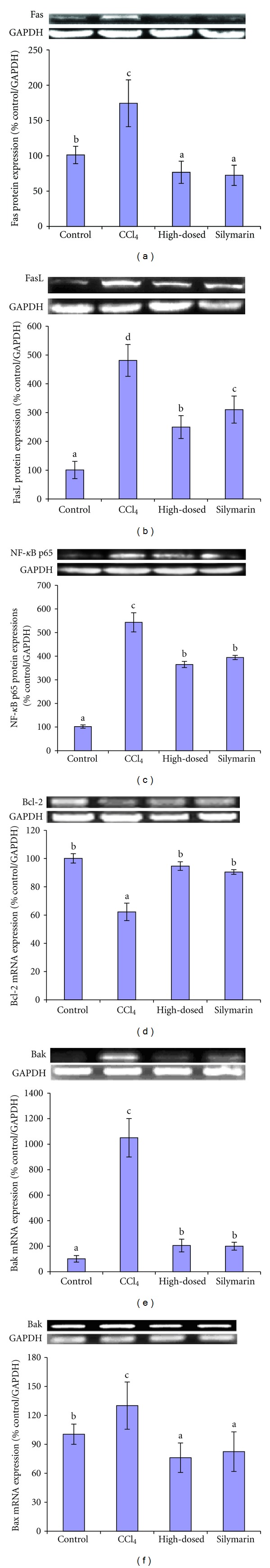
(a–c) Western blot analysis of Fas, FasL, and NF-*κ*B p65 protein expressions in livers of the tested mice. (d–f) RT-PCR analysis of Bcl-2, Bak, and Bax mRNA expressions in livers of the tested mice. Values are given as mean ± SD (*n* = 3); bars with different alphabets differ significantly at *P* < 0.05 level.

In order to evaluate the protective effect of TSAV against CCl_4_-induced acute liver injury in terms of its influence on the Fas/FasL and NF-*κ*B p65, TSAV at high dose was chosen to carry out the next experiments. As seen in Figures 7(a) and 7(b), the expressions of Fas and FasL protein were increased by nearly 2-folds and 5-folds in the liver of CCl_4_-treated mice compared with the normal mice, while their expressions were significantly decreased in TSAV and silymarin mice. The expression of NF-*κ*B p65 was increased by nearly 6-folds in CCl_4_-treated mice and TSAV and silymarin partially prevented this effect (Figure 7(c)). 

### 3.11. RT-PCR Analysis

The expressions of Bcl-2, Bak, and Bax mRNA are shown in Figures 7(d)–7(f). To exclude variations due to RNA quantity and quality, the data were adjusted to GAPDH expression. The levels of Bak and Bax were markedly increased, and the level of Bcl-2 was markedly decreased in the CCl_4_ group compared with the control group, which indicated that the liver in mice was acutely injured. Their levels were significantly reversed in TSAV high-dosed group and silymarin group.

### 3.12. Immunohistochemistry Analysis

**Figure 8:**
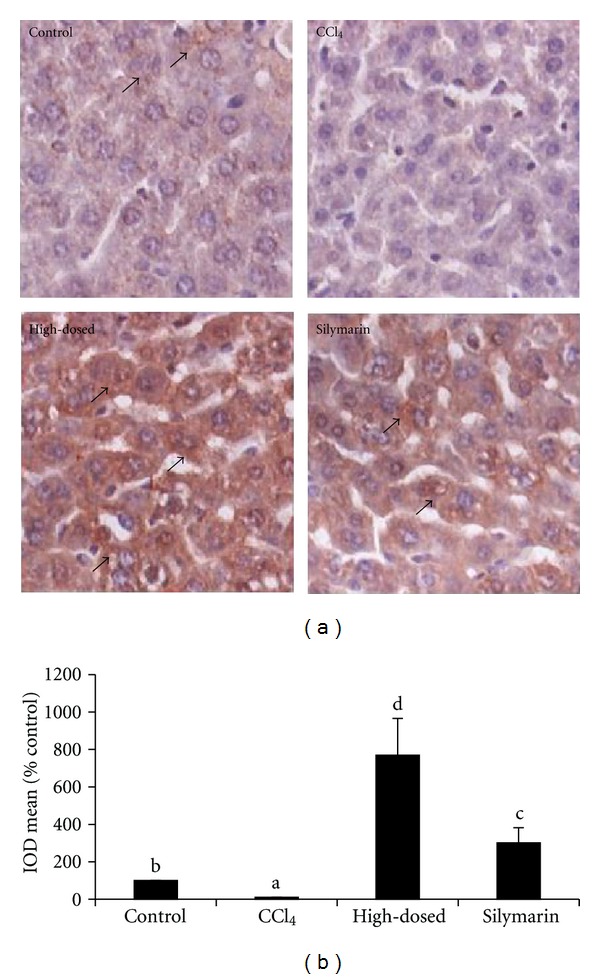
(a) Immunohistochemistry for Bcl-2 in livers of the tested mice. Solid arrows point to positive areas, which are characterized by intense brown nuclear staining. Original magnification: ×400. (b) The results of immunohistochemistry analysis for Bcl-2 in livers of the tested mice. Values are given as mean ± SD (*n* = 3); bars with different alphabets differ significantly at *P* < 0.05 level.

**Figure 9:**
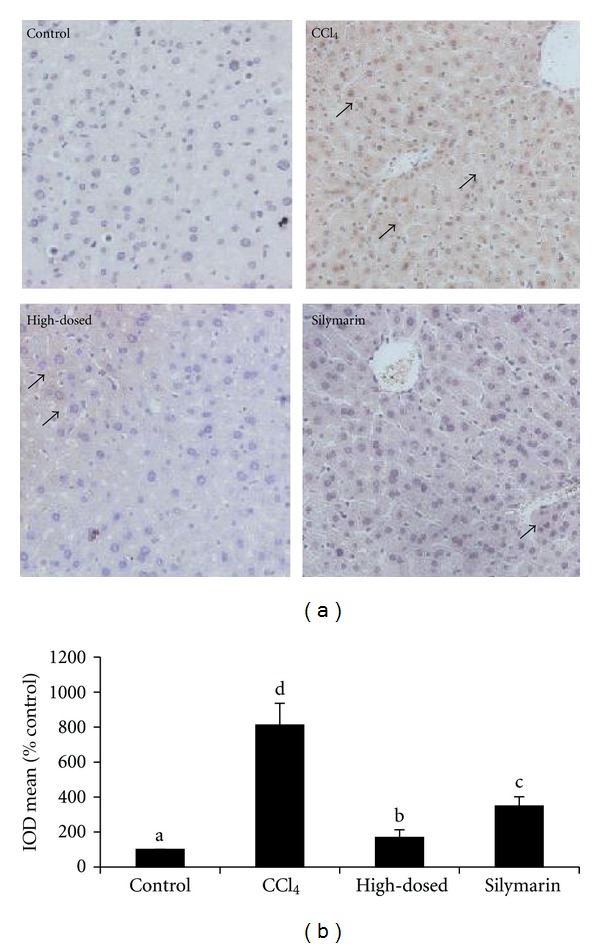
(a) Immunohistochemistry for p53 in livers of the tested mice. Solid arrows point to positive areas, which are characterized by intense brown nuclear staining. Original magnification: ×100. (b) The results of immunohistochemistry analysis for p53 in livers of the tested mice. Values are given as mean ± SD (*n* = 3); bars with different alphabets differ significantly at *P* < 0.05 level.

The immunohistochemistry was applied to analyze the expression of Bcl-2 and p53 proteins. As shown in Figure 8, the expression of Bcl-2 in the CCl_4_ group was 5 times lower compared with the control group. TSAV exhibited opposite effect, and Bcl-2 expression of TSAV at high dose was higher than that of the control group and silymarin group. The expression of p53 (Figure 9) in CCl_4_ group was upregulated compared with the control group (8-folds), and the levels were significantly decreased in TSAV high-dosed group and silymarin group. TSAV at high dose was more effective in upregulating Bcl-2 protein and downregulating p53 protein than those of silymarin.

## 4. Discussion

A number of researches have focused on herbal and plant extracts that possess hepatoprotective activities in recent years [18, 19]. Saponins extracts of plants exert various pharmacological effects to control many diseases, including hepatoprotective activities [20, 21]. The present study is the first to evaluate the potential hepatoprotective effects of TSAV. Our data showed that TASV exhibited antioxidant, anti-inflammatory effects and regulating related apoptosis proteins against CCl_4_-induced acute liver damage.

Serum hepatic enzymes such as AST, ALT, and ALP were employed in the evaluation of hepatic disorders. In CCl_4_-induced acute liver damage models, the levels of ALT, AST, and ALP in serum were significantly suppressed by TSAV, and the inhibitory effects of TSAV (200 mg/kg) on levels of ALT, AST, and ALP in serum were better than those of silymarin (200 mg/kg). Histopathological lesions supported these biochemical results. As shown in Figure 2, hepatocyte and hepatic cord degeneration, focal necrosis, and inflammatory infiltration in mice livers were induced by CCl_4_, which could be effectively ameliorated by TSAV pretreatment.

Lipid peroxide is a major parameter which can be included as a marker of oxidative damage. MDA is widely used as a parameter of lipid peroxidation [22]. In this study, increased liver MDA contents in CCl_4_-treated group suggested that natural antioxidant defense mechanism to scavenge excessive free radicals has been compromised [23, 24]. TSAV at middle and high dose significantly inhibited the formation of MDA in the liver, and their inhibitory effects were better than those of silymarin.

Among the cellular antioxidant enzymes, SOD, CAT, GR, and GPX are important in terms of protecting the liver from CCl_4_-induced damage [25]. SOD and CAT are easily inactivated by lipid peroxides or reactive oxygen species, as reported by this study in CCl_4_-treated group, and there was a significant decrease in hepatic SOD and CAT levels. GR and GPX are glutathione-related enzymes, which can catalyze the synthesis of GSH to ease the burden of lipid peroxidation [25]. In the reaction catalyzed by GPX, GSH is oxidized to GSSG, which can then be reduced back to GSH by GR. In this study, CCl_4_ administration to mice declined antioxidant capacity of the mice liver as evinced in decreased activity of the antioxidant enzymes. TSAV prevented the reduction in the antioxidant enzyme activities and consequent oxidative damage to the liver. GSH significantly decreased, while GSSG augmented in mice treated with CCl_4_ in contrast with the control group, whereas TSAV reversed to normal levels these parameters. Therefore, the GSH/GSSG ratio decreased following CCl_4_ administration, while TSAV and silymarin recovered this ratio partially.

TNF-*α* and IL-6 are pleiotropic cytokines associated with a variety of physiological and pathological conditions [26]. In our study, the serum TNF-*α* and IL-6 levels increased in CCl_4_-treated group, which is in accordance with the finding of Simeonova et al. [27]. TNF-*α* seems to be responsible for regulating products that stimulate inflammation and fibrosis in CCl_4_-induced hepatotoxicity [28]. TSAV pretreatment inhibited the increase of TNF-*α* and IL-6, suggesting TSAV attenuated CCl_4_-induced inflammatory cascade in the liver. Considerable evidence suggested that TNF-*α* and IL-6 contribute to the pathogenesis of liver inflammatory diseases by activating the NF-*κ*B signaling pathway [29]. We found that NF-*κ*B p65 was activated in the CC_4_ group, and this activation was partially prevented by TSAV and silymarin administration, thus decreasing the inflammatory response.

Overproduction of iNOS is associated with inflammatory disorders and the pathophysiology of many disorders, including hepatocarcinoma and autoimmune diseases [30, 31]. TNF-*α* can induce iNOS, stimulate production of nitric oxide, and contribute to nitrosative stress [32]. In this study, increased iNOS level in the serum of CCl_4_-treated mice indicated that enhanced production of NO and nitrosative stress, as a response to liver damage. Our findings suggested that iNOS mediated acute CCl_4_-induced liver damage and its inhibition by TSAV exerted beneficial effects in the prevention of acute hepatic damage.

Caspase-3 and caspase-8 are two caspases proteins which play a central role in the execution-phase of cell apoptosis [33]. Our results showed that after CCl_4_ treatment, considerable caspase-3 and caspase-8 were observed, which inferred that apoptotic effect of CCl_4_ to hepatic cells could be related to the induction of caspase activation. TSAV pretreatment significantly decreased caspase-3 and caspase-8 activities, and TUNEL staining demonstrated that TSAV significantly decreased the number of TUNEL positive cells. In addition, TASV effectively inhibited the DNA ladder induced by CCl_4_. These results indicated that TSAV could restrain the activities of these two caspases and accordingly inhibit hepatic cells apoptosis. 

Cell apoptosis involves at least two major pathways. One is the death receptor pathway. It is known that death receptor-ligand interactions, like the Fas/FasL interaction, are important initiators of apoptosis by the extrinsic pathway [34]. Researches indicated that the suppressions of Fas and FasL protein reduce hepatic cell death associated with liver injury [35, 36]. Moreover, activations of the caspase cascades by the Fas/FasL interaction have been demonstrated to be essential for apoptosis induction in liver injury [34]. In this study, high-dosed TSAV significantly decreased CCl_4_-induced Fas and FasL upregulation, which inferred that the suppression of Fas and FasL protein expression and inhibition of caspase-3 and caspase-8 activities by TSAV played an important role in protecting against CCl_4_-induced acute liver damage. 

The other pathway of cell apoptosis is the mitochondrial pathway, which mainly involves the Bcl-2 family [35]. The release of proapoptotic factors from mitochondria is controlled by Bcl-2 and Bax, which are both members of the Bcl-2 family but have opposing effects on cell life and death [36]. In the present study, compared with CCl_4_-treated group, proapoptotic Bax and Bak mRNA were downregulated, whereas antiapoptotic Bcl-2 mRNA and protein were upregulated in high-dosed TSAV. In addition, Bcl-2 family proteins are known to be subject to regulation by p53 [37, 38], a tumor suppressor protein, and its expression in this study was significantly decreased in high-dosed TSAV pretreated group compared with the CCl_4_ group. These results suggested that TSAV could exert its hepatoprotective effects through the interaction of these proteins.

## 5. Conclusions

In summary, this study demonstrated that TSAV has hepatoprotective effects against CCl_4_-induced acute hepatic damage in mice, which suggested TSAV might be developed to be a potential hepatoprotective drug. Its effects could be associated with increase in antioxidant activities and inhibition of inflammatory mediators as well as regulating apoptosis-related genes. These results will benefit us to further study the exact mechanism of hepatoprotective actions by TSAV.

## Figures and Tables

**Table 1:** Primer sequences of three genes selected for RT-PCR with GAPDH as an internal control.

Gene name	Primers (5′–3′)
Bcl-2	F: ATGTGTGTGGAGAGCGTCAACC
	R: TGAGCAGAGTCTTCAGAGACAGCC

Bax	F: GGCAGCCCCGGCGCGGACCCCTCGGTCA
	R: TAACATCAGTTCCTCTCAGAGGAACCT

Bak	F: ACAAGGACCAGGTCCTCCTAGGC
	R: GCGATGCAATGGTGCAGTATGAT

GAPDH	F: ATGGTGAAGGTCGGTGTGAAC
	R: GTCTTCTGGGTGGCAGTGATG

F: forward primer; R: reverse primer.

**Table 2:** Effects of TSAV on serum enzyme activities with CCl_4_-induced hepatic damage in mice.

Group	AST (IU/L)	ALT (IU/L)	ALP (IU/L)
I	58.71^a^ ± 17.02	24.10^a^ ± 3.72	139.58^a^ ± 15.53
II	1724.82^d^ ± 478.62	4112.02^d^ ± 1062.96	250.12^c^ ± 14.99
III	1250.11^c^ ± 334.93	2583.45^c^ ± 426.52	217.23^b^ ± 23.71
IV	1164.23^c^ ± 259.20	2563.52^c^ ± 401.25	194.98^b^ ± 18.39
V	901.60^b^ ± 231.51	2231.91^b^ ± 407.22	151.08^a^ ± 07.41
VI	1190.54^c^ ± 262.20	2533.93^c^ ± 446.91	166.78^a^ ± 13.84

Group I: control; Group II: CCl_4_; Group III: TSAV 50 mg/kg + CCl_4_; Group IV: TSAV 100 mg/kg + CCl_4_; Group V: TSAV 200 mg/kg + CCl_4_; Group VI: silymarin 200 mg/kg + CCl_4_.

Values are given as mean ± SD (*n* = 10 mice/group); means with different alphabets differ significantly at *P* < 0.05 level.

**Table 3:** Effects of TSAV on liver lipid peroxidation and antioxidant enzymes with CCl_4_-induced hepatic damage in mice.

Group	MDA^A^	SOD^B^	CAT^C^	GPX^D^	GR^E^	GSH^F^	GSSG^G^	GSH/GSSG
I	0.82^a^ ± 0.04	35.71^b^ ± 3.60	382.72^e^ ± 22.79	247.35^d^ ± 42.21	7.56^c^ ± 1.25	6.02^d^ ± 0.55	0.39^a^ ± 0.05	15.75^f^ ± 3.12
II	1.81^c^ ± 0.15	26.12^a^ ± 0.82	218.86^a^ ± 44.95	153.31^a^ ± 26.34	5.12^a^ ± 0.66	4.12^a^ ± 0.79	0.78^d^ ± 0.11	5.40^a^ ± 1.28
III	1.69^c^ ± 0.22	29.16^a^ ± 2.50	241.06^b^ ± 36.12	180.66^b^ ± 24.72	5.87^b^ ± 0.93	4.62^b^ ± 0.87	0.68^c^ ± 0.14	6.84^b^ ± 0.98
IV	1.13^b^ ± 0.11	35.32^b^ ± 3.13	252.74^b^ ± 32.43	207.92^c^ ± 18.37	6.24^b^ ± 1.02	4.88^b^ ± 0.76	0.64^c^ ± 0.05	7.65^c^ ± 0.81
V	0.93^a^ ± 0.08	42.87^c^ ± 3.61	324.78^d^ ± 25.19	236.84^d^ ± 29.52	7.31^c^ ± 1.21	5.64^c^ ± 0.47	0.53^b^ ± 0.09	10.96^e^ ± 2.08
VI	1.25^b^ ± 0.18	32.04^b^ ± 2.05	289.45^c^ ± 33.94	230.11^d^ ± 21.26	7.24^c^ ± 0.82	5.34^c^ ± 0.38	0.58^b^ ± 0.06	9.25^d^ ± 1.10

Group I: control; Group II: CCl_4_; Group III: TSAV 50 mg/kg + CCl_4_; Group IV: TSAV 100 mg/kg + CCl_4_; Group V: TSAV 200 mg/kg + CCl_4_; Group VI: silymarin 200 mg/kg + CCl_4_.

^A^nmol/mg protein, ^B^U/mg protein, ^C^U/g protein, ^D^U/mg protein,^ E^U/g protein,^F,G^ *μ*mol/g protein. Values are given as mean ± SD (*n* = 10 mice/group); means with different alphabets differ significantly at *P* < 0.05 level.
